# Luhong Formula Has a Cardioprotective Effect on Left Ventricular Remodeling in Pressure-Overloaded Rats

**DOI:** 10.1155/2020/4095967

**Published:** 2020-05-30

**Authors:** Qian Liu, Hui-Yan Qu, Hua Zhou, Jing-Feng Rong, Tian-Shu Yang, Ji-Jie Xu, Xiao-Li Yang, Zhen-Zhen Lan

**Affiliations:** ^1^Cardiovascular Department of Traditional Chinese Medicine, Shuguang Hospital Affiliated to Shanghai University of Traditional Chinese Medicine, Shanghai 201203, China; ^2^Cardiovascular Research Institute of Traditional Chinese Medicine, Shuguang Hospital Affiliated to Shanghai University of Traditional Chinese Medicine, Shanghai 201203, China

## Abstract

**Background:**

Luhong formula (LHF)—a traditional Chinese medicine containing Cervus nippon Temminck, Carthamus tinctorius L., Astragalus membranaceus (Fisch.) Bge. var. mongholicus (Bge.) Hsiao, Codonopsis pilosula (Franch.) Nannf., Cinnamomum cassia Presl, and Lepidium apetalum Willd—is used in the treatment of heart failure, but little is known about its mechanism of action. We have investigated the effects of LHF on antifibrosis.

**Methods:**

Forty-eight SD male rats were randomly assigned into six groups (*n* = 8), model group, sham-operation group, perindopril group (0.036 mg/ml), LHF high doses (LHF-H, 1.44 g/mL), LHF middle doses (LHF-M, 0.72 g/mL), and LHF low doses (LHF-L, 0.36 g/mL). Except the sham-operation group, the other groups were received an abdominal aorta constriction to establish a model of myocardial hypertrophy. The HW and LVW were measured to calculate the LVW/BW and HW/BW. ELISA was used to detect the serum concentration of BNP. The expressions of eNOS, TGF-*β*1, caspase-3, VEGF, and VEGFR2 in heart tissues were assessed by western blot analysis. mRNA expressions of eNOS, Col1a1, Col3a1, TGF-*β*1, VEGF, and VEGFR2 in heart tissues were measured by RT-PCR. The specimens were stained with hematoxylin-eosin (HE) and picrosirius red staining for observing the morphological characteristics and collagen fibers I and III of the myocardium under a light microscope.

**Results:**

LHF significantly lowered the rat's HW/BW and LVM/BW, and the level of BNP in the LHF-treated group compared with the model group. Histopathological and pathomorphological changes of collagen fibers I and III showed that LHF inhibited myocardial fibrosis in heart failure rats. Treatment with LHF upregulated eNOS expression in heart tissue and downregulated Col1a1, Col3a1, TGF-*β*1, caspase-3, VEGF, and VEGFR2 expression.

**Conclusion:**

LHF can improve left ventricular remodeling in a pressure-overloaded heart failure rat model; this cardiac protective ability may be due to cardiac fibrosis and attenuated apoptosis. Upregulated eNOS expression and downregulated Col1a1, Col3a1, TGF-*β*1, caspase-3, VEGF, and VEGFR2 expression may play a role in the observed LHF cardioprotective effect.

## 1. Introduction

Heart failure (HF) has a range of clinical syndromes characterized by decreased cardiac function; it is the final destination of most cardiovascular diseases. HF afflicts millions of patients annually. The incidence of HF is high, and its prognosis is poor. HF is a severe public problem in China. At present, cardiovascular disease is the leading cause of death among Chinese urban and rural residents [1]. An epidemiological survey showed that the prevalence rate of HF in adults was 0.9% [2]. Hypertension (HTN) is one of the most common cardiovascular diseases and affects 972 million people worldwide [3]. In addition, HTN is one of the most common antecedent conditions in patients with HF. In a cardiovascular health study, the proportion of HTN with HF was 82% among selected comorbidities [4]. In the China Heart Failure Registry Study, there were 4649 (54.6%) HF patients with HTN among a total of 8516 HF patients [5]. Therefore, it is of great significance to study the treatment of heart failure caused by hypertension. Due to long-time raised blood pressure, HTN will eventually lead to cardiac dysfunction with left ventricular hypertrophy, and myocardial fibrosis and further promote ventricular remodeling. Myocardial fibrosis is the main pathological change in HF. With the onset of HF, collagen deposition enhances and leads to fibrosis. Recent studies have demonstrated that there are many classical factors and pathway that contribute to the regulation of myocardial fibrosis and ventricular remodeling, such as eNOS, Col1a1, Col3a1, TGF-*β*1, and VEGF/VEGFR2 pathway [8, 9]. However, no effective antifibrotic agents are currently available to interrupt specific aspects of this pathway. Identification of signaling pathways and proteins that regulate hypertrophic growth and fibrosis will provide useful information for therapy aimed at preventing the development of HF.

In China, traditional Chinese medicine (TCM) is an efficient treatment for HF, and the category of HF belongs to “heart-kidney Yang deficiency, blood stasis” [8]. Luhong formula (LHF, Chinese national patent number ZL2006101473050) is an empirical compound prescription of several common medicinal herbs in TCM. In a multicenter clinical study (Chinese Clinical Trial Registration Number ChiCTR-TRC-13003242) of LHF, the HF treatment demonstrated a superior performance after a 3-month follow-up with respect to left ventricular ejection fraction (LVEF), fractional shortening (FS), stroke volume (SV), New York Heart Association functional classification, Minnesota quality of life scores, and a 6-minute walk distance test [9]. Recent studies have shown that LHF produces a cardiac protection effect through a variety of pathways involved in the anti-renin-angiotensin system (RASS), antifibrosis, and myocardial metabolism. LHF has been shown to significantly increase the plasma ACE2-Ang (1–7) axis and reduce Ang II levels in rats with heart failure [10]. In addition, LHF inhibits myocardial fibrosis in a paracrine manner by activating the gp130/JAK2/STAT3 pathway in cardiomyocytes [11]. Col1a1 and Col3a1 encode predominant components of cardiac collagens. TGF-*β*1 affects the expression of collagen I and collagen III, which results in cardiac interstitial fibrosis. VEGF/VEGFR2 pathway is involved in cardiomyocyte hypertrophy and promotes ventricular remodeling. Enhanced eNOS activity and increased NO levels could inhibit cardiac remodeling caused by HF. They are all classical factors and pathway that contribute to the regulation of myocardial fibrosis and ventricular remodeling. Caspase-3 is an important protein related to cardiomyocyte apoptosis. It is not clear whether LHF can inhibit myocardial fibrosis and attenuate apoptosis by other ways such as these classic factors and pathway. In this study, we first investigated the antifibrotic efficacy of LHF in heart failure rats induced by abdominal aorta constriction. We then determined the mechanisms behind the cardioprotective effect of LHF by examining its effects on eNOS, Col1a1, Col3a1, TGF-*β*1, caspase-3, and VEGF/VEGFR2 pathway, and the relationship between them.

## 2. Methods

### 2.1. Materials

Perindopril (an ACE inhibitor) was purchased from Servier (Tianjin) Pharmaceutical Co., Ltd. (China), and BNP detection kits were purchased from Nanjing Jiancheng Bioengineering Institute (China). Reverse transcriptase (RT) kits were purchased from Thermo Fisher Scientific Inc. (Waltham, Massachusetts, USA). Bicinchoninic acid (BCA) kits were purchased from Thermo Fisher Scientific Inc. (Waltham, Massachusetts, USA).

### 2.2. Preparation of Luhong Formula

The formula to create one dose of Luhong is presented in Table 1. Luhong formula is composed of 9 g of Cervus nippon Temminck (lot number 2012102601), 9 g of Carthamus tinctorius L. (lot number 13011401), 30 g of Astragalus membranaceus (Fisch.) Bge. var. mongholicus (Bge.) Hsiao (lot number 2013011504), 30 g of Codonopsis pilosula (Franch.) Nannf. (lot number 2012122701), 9 g of Cinnamomum cassia Presl (lot number 2012120212), and 20 g of Lepidium apetalum Willd (lot number 11111401) [11]. All herbs were manufactured according to the Pharmacopoeia of the People's Republic of China (2010). All herbs, except Cervus nippon Temminck buckhorn, were added to 10 times the amount of water, soaked for 30 minutes, decocted for 45 minutes (100°C), and filtered by a No. 6 sieve, and then the filtrate was collected. The residue from the herbs continued to be added to 8 times the amount of water, decocted for 30 minutes (100°C), filtered, and collected. The decoctions were combined, decompressed, and concentrated to a relative density of 1.02–1.07 (60 ± 2°C). After cooling to room temperature, the decoctions were centrifuged at high speed (10000 r/min, 10 minutes). Powdered Cervus nippon Temminck buckhorn (over 100 mesh screens) and 10% dextrin were mixed and added to the supernatant fluid after centrifugation. Finally, spray drying was used to dry and extract the granule (1 g of the LHF granule contained 7.92 g of herbal mixtures). The LHF granule was supplied by Original Technology of Shanghai University of Traditional Chinese Medicine. The granule was dissolved in water before use with a crude drug concentration of 0.72 g/ml.

### 2.3. Perindopril

Perindopril tablets (4 mg/tablet) were used in the present study. Perindopril tablets (National medicine permission number H20034053) were purchased from Servier (Tianjin) Pharmaceutical Co., Ltd. (China). The tablets were crushed, subjected to a pharmacopoeia 100 mesh sieve, and added to double-distilled water. Finally, perindopril was formulated to a 0.036 mg/ml concentration.

### 2.4. Animal Model and Administration

Male Sprague Dawley rats (body weight 200–230 g) were provided by the Animal Experiment Center of Shanghai University of Traditional Chinese Medicine. All animal experimental protocols were performed in accordance with the Guide for the Care and Use of Laboratory Animals published by the National Institutes of Health (NIH Publications No. 85-23, revised 1996) and with approval from the Animal Ethics Committee of Shanghai University of Traditional Chinese Medicine (number SZY2013037). All rats were raised in the Animal Experimental Center of Shanghai University of Traditional Chinese Medicine. The temperature of the feeding room was kept at 20°C∼25°C. The relative humidity was between 50%∼65%. The feeding environment was quiet and no noise was allowed. The closed feeding room adopted a light timing device to provide appropriate (12 h light, 12 h dark) day and night light change cycle. The heart failure model was established by abdominal aorta constriction (AAC) as previously described [12]; 54 rats were included in the present study. Briefly, rats were anesthetized with pentobarbital sodium (45 mg/kg) by intraperitoneal injection. The abdominal aorta was dissected above the two renal arteries. A puncture needle (0.7 mm outer diameter) was placed on the abdominal aorta and ligated together with thread 4. Then, the puncture needle was drawn out and the ligated artery was partially ligated with 60%–70% degree of constriction. After 4 weeks, the systolic blood pressure was >140 mmHg, indicating that the hypertension model was successful [13]. A total of 11% of the rats died during the experiment, and the remaining 48 surviving rats were randomly assigned to the following groups: model group; sham-operation group; perindopril group; and high-dose (LHF-H), middle-dose (LHF-M), and low-dose (LHF-L) LHF groups, with 8 rats in each group. The sham-operation group was used as the control group without HF, where rats underwent a similar procedure but without actual ligation of the abdominal aorta. Rats 4 weeks followed the procedure were regarded as HF. From the fifth week, the model group and sham-operation group were treated with drinking water; the perindopril group was treated with perindopril; the high, middle, and low dose of LHF groups were treated with high, middle, and low doses of LHF, respectively. According to the human and animal surface area of the equivalent dose conversion ratio table, the concentrations of LHF in high, middle, and low doses of LHF groups were 1.44 g/mL, 0.72 g/mL, and 0.36 g/mL, respectively. Rats were given by gavage once a day, 6 times a week. All animals were intragastrically administered by intragastric syringe and fed under the same condition for 8 weeks. The head of the rat was fixed by grasping the skin of the back and neck behind the two ears of the rat with thumb and index finger of the left hand. The syringe was taken with the right hand and the needle was inserted into the pharynx from the left corner of the mouth of the rat. Along the back wall of the upper jaw, the front end of the needle was moved gently into the esophagus without any sense of conflict, and then the needle was inserted into the stomach and the needle core was pushed, and the test substance was injected. During treatment, 1 rat in the model group died of heart failure; 1 rat in the perindopril group died of heart failure; 1 rat in the middle-dose (LHF-M) LHF group died of heart failure; 1 rat in the low-dose (LHF-L) LHF group died of heart failure; 1 rat in the low-dose (LHF-L) LHF group died of improper gavage. There were 43 rats at the end of the treatment.

### 2.5. Heart Weight Index and Left Ventricular Mass Index

After treatment for 8 weeks, the animals were anesthetized to collect blood from the abdominal aorta, and the heart was rapidly removed and rinsed with cold physiological saline, and water was absorbed by filter paper. The excess tissue, such as the pericardium and vessels around the hearts, was removed, and water was absorbed by filter paper. After the hearts were weighed on a balance, the left ventricular sections were taken from the whole hearts to weigh and then immediately put into liquid nitrogen for storage. The whole heart weight (HW) and left ventricular weight (LVW) were measured to calculate the left ventricular mass index (LVW/BW) and heart weight index (HW/BW) with the following formula (Table 1):

(1) $$\begin{array}{c} \text{Heart weight index}=\frac{\text{heart weight}\left(\text{HW}\right)}{\text{body weight}\left(\text{BW}\right)},\\ \text{Left ventricular mass index}=\frac{\text{left ventricular weight}\left(\text{LVW}\right)}{\text{body weight}\left(\text{BW}\right)}. \end{array}$$

### 2.6. Serum Brain Natriuretic Peptide

Blood samples were collected from the abdominal aorta and let stand for 2 hours. The serum was collected after centrifugation. Biotin double antibody sandwich enzyme-linked immunosorbent assay (ELISA) was used to detect the serum concentration of brain natriuretic peptide (BNP). The absorbance (optical density, OD) was read through an enzymatic reader at 450 nm. The concentration of BNP in the samples was calculated by standard curve (Table 2).

### 2.7. Western Blot Analysis

The expressions of eNOS, TGF-*β*1, caspase-3, VEGF, and VEGFR2 in heart tissues were assessed by western blot analysis. Briefly, myocardial tissue was cut into tiny fragments. A total of 150–250 *μ*l of lysate was added to each 20 mg tissue, and homogenates were homogenized until completely lysed. The supernatant was collected after centrifugation (4°C, 12000 rpm for 15 min). The proteins were extracted, separated, and transferred onto polyvinylidene difluoride (PVDF) membranes. The membranes were blocked with 5% skimmed milk powder overnight at 4°C. The blots were reacted with antibodies to eNOS (1 : 500, Abcam, Ab50010), TGF-*β*1 (1 : 800, Abcam, Ab92486), caspase-3 (1 : 500, CST, 14220T), VEGF(1 : 1000, Abcam, Ab46154), and VEGFR2 (1 : 1000, Abcam, Ab11939) overnight at 4°C and then with secondary antibody at 37°C for 1 hour. The grayscale signals of the blots were visualized and then analyzed using image analysis.

### 2.8. Real-Time PCR

mRNA expressions of eNOS, Col1a1, Col3a1, TGF-*β*1, VEGF, and VEGFR2 in heart tissues were measured by quantitative real-time polymerase chain reaction (RT-PCR). Total RNA was isolated with Trizol reagent (Invitrogen, USA, 1596-026). PCR was performed under the following conditions: elimination of DNA in total RNA was at 37°C for 30 min, and then EDTA (1 *μ*L) was held at 65°C for 10 min. Reverse transcription reaction program was performed at 37°C for 60 min, 85°C for 5 min, and 4°C for 5 min. The prepared cDNA was amplified by PCR at 95°C for 10 min, followed by 40 cycles at 95°C for 15 s and 60°C for 45 s. Then, the reaction procedure was at 95°C for 15 s, 60°C for 1 min, 95°C for 15 s, and 60°C for 15 s. We used the GAPDH gene as an internal reference and the ΔCT method to analyze the results. The primer sequences are shown in Table 3.

### 2.9. Histomorphology Observation

**Figure 1:**
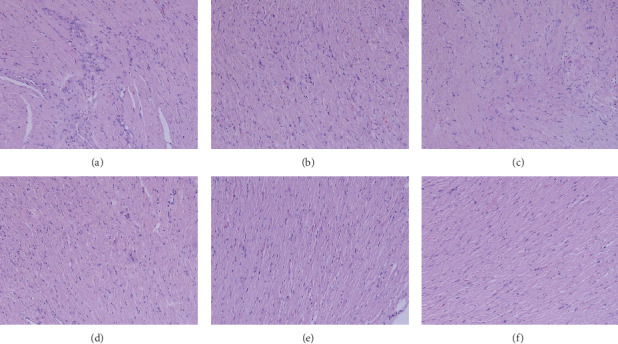
Histomorphology observation of representative images showing myocardial cells and interstitial fibrosis (HE staining, 100×). (a) Model group; (b) LHF-L group; (c) LHF-M group; (d) perindopril group; (e) LHF-H group; (f) sham group.

**Figure 2:**
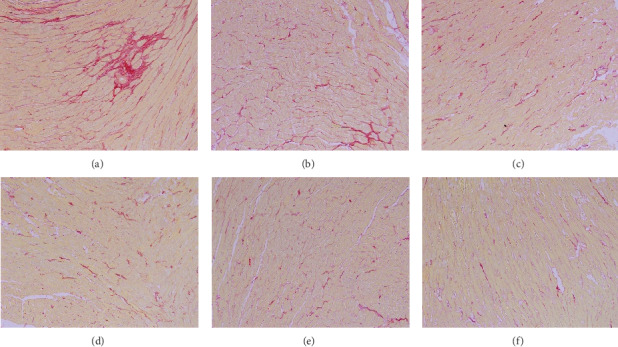
Histomorphology observation of representative images showing myocardial cells and interstitial fibrosis (picrosirius red staining, 100×). (a) Model group; (b) LHF-L group; (c) LHF-M group; (d) perindopril group; (e) LHF-H group; (f) sham group.

**Figure 3:**
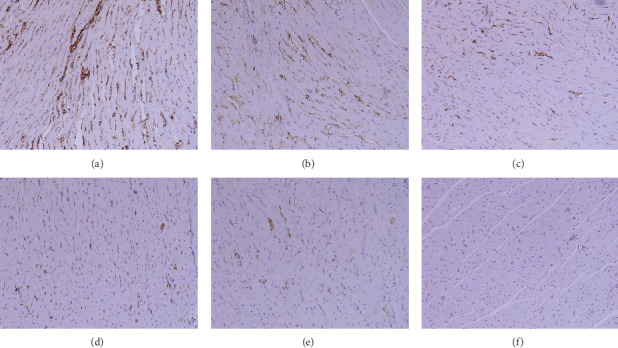
Histomorphology observation of representative images showing myocardial cells and interstitial fibrosis (collagen fibers I, 100×). (a) Model group; (b) LHF-L group; (c) LHF-M group; (d) perindopril group; (e) LHF-H group; (f) sham group.

**Figure 4:**
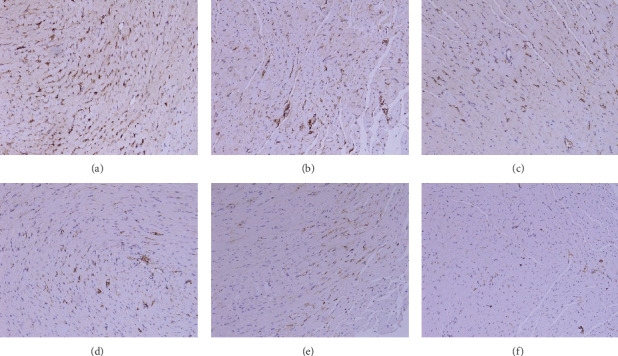
Histomorphology observation of representative images showing myocardial cells and interstitial fibrosis (collagen fibers III, 100×). (a) Model group; (b) LHF-L group; (c) LHF-M group; (d) perindopril group; (e) LHF-H group; (f) sham group.

The left ventricular myocardium and intraventricular septum were separated, fixed with 10% formaldehyde solution, made in coronal sections, embedded in paraffin, and cut into slices. Finally, the specimens were stained with hematoxylin-eosin (HE) and picrosirius red staining for observing the morphological characteristics and collagen fibers I and III of the myocardium under a light microscope. The detection was performed by the pathology department of Shuguang Hospital (Figures 1–4).

### 2.10. Statistical Analysis

Statistical testing was performed with SPSS 21.0. The results are expressed as means ± standarddeviation ($\bar{x}\pm s$). One-way ANOVA test was used when the analysis between groups accorded with normality and homogeneity of variance. LSD of post hoc statistical test was used after ANOVA. LSD was used to compare groups with each other. Rank sum test of two independent samples was used when the analysis between groups did not conform to the normality and homogeneity of variance. *P* values of less than 0.05 were considered statistically significant.

## 3. Results

### 3.1. LHF Improved LV Remodeling in HF Rats

HW/BW and LVW/BW were significantly lower in each LHF group and perindopril group than in the model group (*P* < 0.01), but higher than in the sham group (*P* < 0.01). The perindopril group exhibited lower HW/BW and LVW/BW compared with each LHF group (*P* < 0.01). HW/BW in the LHF-H group was lower than that in the LHF-M group (*P* < 0.05). There was no statistically significant difference between LHF-H, LHF-M, and LHF-L groups about LVW/BW (Table 1).

### 3.2. Effect on Brain Natriuretic Peptide (BNP)

BNP was significantly higher in the model group, perindopril group, LHF-H group, LHF-M group, and LHF-L group compared with the sham group (*P* < 0.01). All LHF groups and the perindopril group effectively reduced BNP in rats with HF compared with the model group (*P* < 0.01). LHF-H group reduced BNP more effectively than LHF-M (*P* < 0.05) and LHF-L (*P* < 0.01) groups (Table 2).

### 3.3. Protein Expression in HF Rat Hypertrophic Cardiomyocytes and Effects of LHF

**Figure 5:**
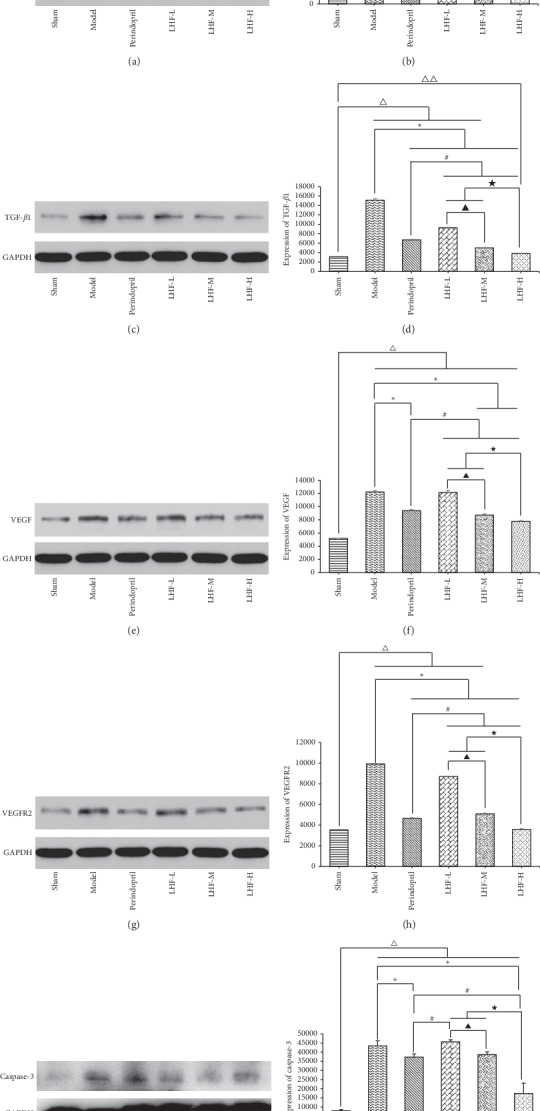
Protein expression of myocardial tissue in pressure-overloaded heart failure rats. (a) Western blot analysis of eNOS protein expression in myocardial tissue. (b) Quantification of eNOS protein expression in myocardial tissue by densitometry. (c) Western blot analysis of TGF-*β*1 protein expression in myocardial tissue. (d) Quantification of TGF-*β*1 protein expression in myocardial tissue by densitometry. (e) Western blot analysis of VEGF protein expression in myocardial tissue. (f) Quantification of VEGF protein expression in myocardial tissue by densitometry. (g) Western blot analysis of VEGFR2 protein expression in myocardial tissue. (h) Quantification of VEGFR2 protein expression in myocardial tissue by densitometry. (i) Western blot analysis of caspase-3 protein expression in myocardial tissue. (j) Quantification of caspase-3 protein expression in myocardial tissue by densitometry. Data are expressed as mean ± standard deviation (SD). ^Δ^*P* < 0.01, ^ΔΔ^*P* < 0.05 compared with sham; ^*∗*^*P* < 0.01, ^*∗∗*^*P* < 0.05 compared with model; ^#^*P* < 0.01 compared with perindopril; ^★^*P* < 0.01 compared with LHF-H; ^▲^*P* < 0.01, ^▲▲^*P* < 0.05 compared with LHF-M.

The protein expression levels of eNOS, TGF-*β*1, caspase-3, VEGF, and VEGFR2 were detected by western blot analysis (Figures 5(a), 5(c), 5(e), 5(g), and 5(i)). The expression level of eNOS was significantly lower in the model group, perindopril group, LHF-H group, LHF-M group, and LHF-L group compared with the sham group (*P* < 0.01), and the TGF-*β*1 and VEGF protein levels were significantly higher in the model group, perindopril group, LHF-H group, LHF-M group, and LHF-L group compared with the sham group (*P* < 0.01). The VEGFR2 protein level was significantly higher in the model group, perindopril group, LHF-M group, and LHF-L group compared with the sham group (*P* < 0.01). The expression levels of eNOS, TGF-*β*1, and VEGFR2 protein were significantly different in each LHF group and perindopril group compared with the model group (*P* < 0.01); the expression levels of VEGF protein were significantly different in the LHF-H group, LHF-M group, and perindopril group compared with the model group (*P* < 0.01). The expression level of eNOS protein was lower in the LHF-M group and LHF-L group compared with the LHF-H group (*P* < 0.01); the expression levels of TGF-*β*1, VEGF, and VEGFR2 protein were higher in the LHF-M group and LHF-L group compared with the LHF-H group (*P* < 0.01). The expression level of caspase-3 protein was higher in the model group, perindopril group, LHF-H group, LHF-M group, and LHF-L group compared with the sham group (*P* < 0.01). The expression level of caspase-3 protein was significantly lower in the LHF-H group compared with the model group (*P* < 0.01) and lower in the perindopril group compared with the model group (*P* < 0.05). The expression levels of caspase-3 protein were higher in the LHF-M group and LHF-L group compared with LHF-H group (*P* < 0.01) (Tables 4 and 5; Figures 5(b), 5(d), 5(f), 5(h), and 5(j)).

### 3.4. mRNA Expression in HF Rat Hypertrophic Cardiomyocytes and Effects of LHF

**Figure 6:**
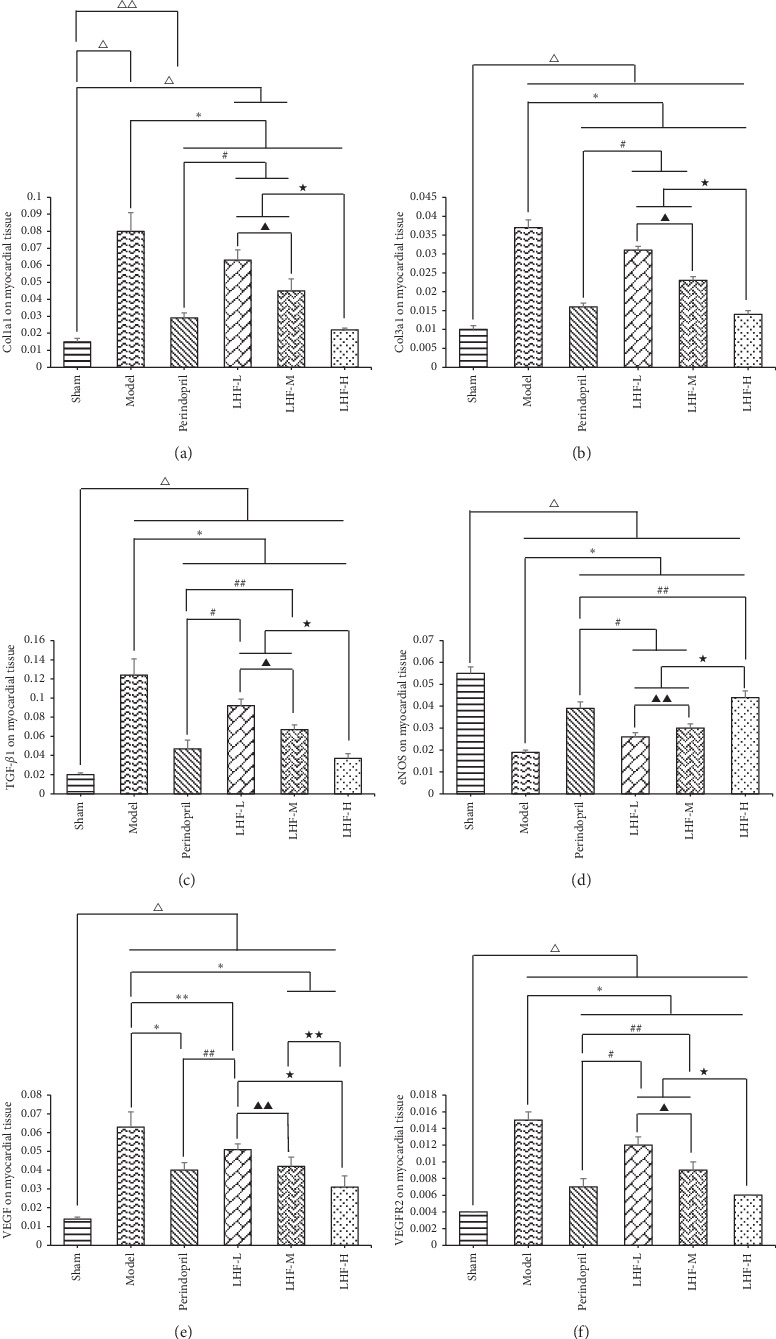
mRNA expression of myocardial tissue in pressure-overloaded heart failure rats. (a) Real-time PCR of COL1a1 mRNA expression in myocardial tissue. (b) Real-time PCR of COL3a1 mRNA expression in myocardial tissue. (c) Real-time PCR of TGF-*β*1 mRNA expression in myocardial tissue. (d) Real-time PCR of eNOS mRNA expression in myocardial tissue. (e) Real-time PCR of VEGF mRNA expression in myocardial tissue. (f) Real-time PCR of VEGFR2 mRNA expression in myocardial tissue. Data are expressed as mean ± standard deviation (SD). ^Δ^*P* < 0.01, ^ΔΔ^*P* < 0.05 compared with sham; ^*∗*^*P* < 0.01, ^*∗∗*^*P* < 0.05 compared with model; ^#^*P* < 0.01, ^##^*P* < 0.05 compared with perindopril; ^★^*P* < 0.01, ^★★^*P* < 0.05 compared with LHF-H; ^▲^*P* < 0.01, ^▲▲^*P* < 0.05 compared with LHF-M.

Real-time PCR was used to examine the mRNA expression of COL1a1, COL3a1, eNOS, TGF-*β*1, VEGF, and VEGFR2. eNOS expression was significantly lower in the model group, perindopril group, LHF-H group, LHF-M group, and LHF-L group compared with the sham group (*P* < 0.01). COL3a1, VEGF, and VEGFR2 protein levels were significantly higher in the model group, perindopril group, LHF-H group, LHF-M group, and LHF-L group compared with the sham group (*P* < 0.01). The expression levels of COL1a1, COL3a1, eNOS, TGF-*β*1, and VEGFR2 protein were significantly different in each LHF group and perindopril group compared with the model group (*P* < 0.01). The expression level of VEGF protein was significantly different in the LHF-H group, LHF-M group, and perindopril group compared with the model group (*P* < 0.01) and different in the LHF-L group compared with the model group (*P* < 0.05). The expression level of eNOS protein was significantly lower in the LHF-M group and LHF-L group compared with the LHF-H group (*P* < 0.01); the expression levels of COL1a1, COL3a1, TGF-*β*1, and VEGFR2 protein were significantly higher in the LHF-M group and LHF-L group compared with the LHF-H group (*P* < 0.01). The expression level of VEGF protein was significantly higher in the LHF-L group compared with the LHF-H group (*P* < 0.01) and higher in the LHF-M group compared with the LHF-H (*P* < 0.05) (Table 6; Figures 6(a)–6(f)).

### 3.5. Effect on Myocardial Fibrosis

The sham group exhibited normal myocardial cells, and the myocardial fibers were arranged in order and the HE or picrosirius red staining was uniform. The model group showed swollen myocardial cells, hyperplastic collagen fibers of the myocardium, and infiltration of inflammatory cells. The myocardial cells in the model group showed abnormalities compared with the sham group, while each LHF group and perindopril group were better than the model group. LHF-H group was more potent in shrinking swollen cells, reducing inflammatory cell invasion, and preventing collagen hyperplasia compared with the perindopril group (Figures 1 and 2).

There were few brown and yellow I, III collagen fibers in the sham group. A large number of brown and yellow I, III collagen fibers with irregular arrangements were observed in the model group. Compared with the model group, collagen fibers were reduced in each LHF group and perindopril group and however showed abnormalities compared with the sham group. LHF-H group was more potent in reducing the number of I, III collagen fibers in the myocardium and improving the disorder of the permutation compared with perindopril (Figures 3 and 4).

## 4. Discussion

**Figure 7:**
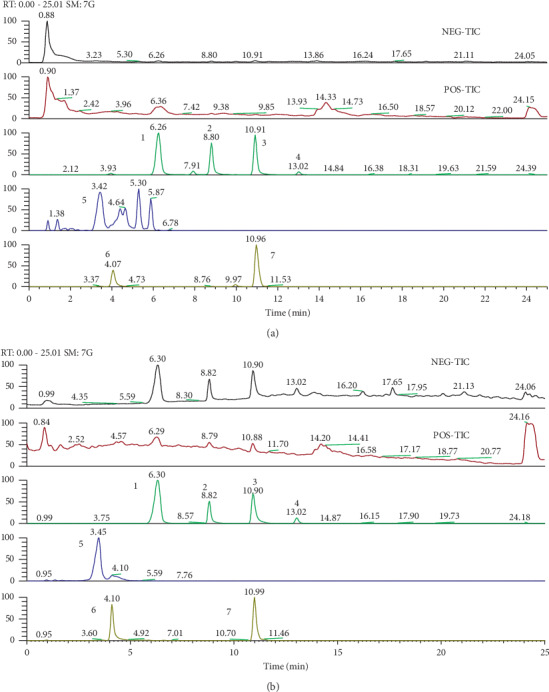
Total-ion chromatograms (TIC) and select-ion chromatograms of LHF extracts samples, (a) the mix standard solution, 1.25 *μ*g/ml; (b) the sample solution, 8.00 mg/ml (stationary phase: ACQUITY UPLC HSS T3 (2.1 mm × 100  mm, 1.8 *μ*m); mobile phase: acetonitrile (a) and water (b) in gradient (time, min/B%: 0/95, 25/5); flow rate: 0.3 mL/min). Peak no: (1) cinnamic acid; (2) codonolactone; (3) formononetin; (4) calycosin; (5) hydroxysafflor yellow A; (6) quercetin-3-O-b-D-glucose-7-O-b-D-gentiobiosiden; (7) astragaloside.

LHF is a TCM that was developed according to TCM theory. Pharmacological studies have found that LHF contains a number of active substances, such as hydroxysafflor yellow A, astragaloside, cinnamic acid, codonolactone, quercetin-3-O-b-D-glucose-7-O-b-D-gentiobiosiden, formononetin, and calycosin [11]. For quality control, the fingerprint spectrum for LHF was performed by UHPLC-Q Exactive system (Thermo, San Jose, CA, USA) (Figure 7). LV remodeling after hypertension contributes to HF. Beneficial effects of LHF on LV remodeling have been observed in patients with HF [9]. Our study demonstrated that LHF could improve cardiac remodeling by inhibiting fibrosis-related gene expression.

HW/BW reflects cardiac hypertrophy, and LVM/BW especially reflects LV hypertrophy. Cardiac hypertrophy, only when accompanied by fibrosis during remodeling, can lead to HF [14]. Our data showed that HW/BW and LVM/BW decreased in each group of LHF and the perindopril group compared with the model group. Therefore, our results showed that LHF could inhibit LV remodeling in rats with HF.

BNP is a classic and highly sensitive marker of myocardial dysfunction, which is released by the heart ventricles in response to changes in the ventricular pressure and/or volume. A previous study has shown that an increase in BNP is associated with HF [15]. An increase in BNP expression has been connected to the remodeling process of the LV [16]. Each LHF group (especially LHF-H group) and the perindopril group showed a significantly reduced level of BNP in rats with HF. Therefore, LHF could improve cardiac function in pressure-overloaded HF rats.

Caspase-3 is an important protein related to cardiomyocyte apoptosis. The activation of caspase-3 is reported in the process of cardiomyocyte apoptosis [17]. The LHF-H group and LHF-M group (especially LHF-H group) and the perindopril group showed a significantly reduced level of caspase-3 in rats with HF. We found that LHF (especially LHF-H group) could attenuate apoptosis through downregulated caspase-3 expression.

eNOS is expressed in cardiac myocytes and plays an important role in the cardiovascular system. eNOS is an important signaling molecule involved in the production of endogenous nitric oxide (NO) [18]. Endogenous NO exhibits an antimyocardial hypertrophy effect and prevents ventricular remodeling [19]. Enhanced eNOS activity and increased NO levels could inhibit cardiac remodeling caused by HF. Real-time PCR and western blot assays revealed that eNOS was significantly elevated in each LHF group (especially LHF-H group) and perindopril group compared with the model group. We found that LHF could prevent ventricular remodeling by enhancing eNOS activity.

Deposition of collagen fibers in the myocardial interstitium is a landmark for the remodeling process seen in a variety of cardiovascular diseases of different etiology. In the pressure-overloaded heart, interstitial fibrosis caused cardiac dysfunction [20]. Col1a1 and Col3a1 are specifically expressed in fibroblasts and encode predominant components of cardiac collagens [21]. Our results showed that compared with the model group, Col1a1 and Col3a1 expression decreased in each LHF group (especially LHF-H group) and the perindopril group. These findings revealed that LHF could alleviate interstitial fibrosis through decreasing the expression of Col1a1 and Col3al in rat myocardial tissue.

TGF-*β*1 is a generated cytokine and plays an important role in fibroblast proliferation, especially in fibronectin and collagen. There is growing evidence that TGF-*β*1 is involved in cardiac fibrosis [22], and overexpression of TGF-*β*1 results in cardiac interstitial fibrosis. In recent years, many studies have confirmed that suppression of TGF-*β*1 can inhibit cardiac fibrosis [23, 24]. TGF-*β*1 is expressed in cardiac myocytes and fibroblasts and can increase type I, III collagen and fibronectin mRNA and protein expression. Each LHF group (especially LHF-H group) and the perindopril group significantly suppressed TGF-*β*1 expression in the LV myocardium compared with the model group.

Fibrillar collagen types I and III are the predominant components of the cardiac extracellular matrix (ECM), which is related to myocardial fibrosis. Studies have shown that TGF-*β*1 affects the expression of collagen I and collagen III [25]. The content of type I collagen determines the stiffness of the myocardium, and the content of type III collagen determines the compliance of the myocardium. Through the histomorphology observation of myocardial cells and interstitial fibrosis, we found that LHF could inhibit the proliferation of collagen. A pathological change of myocardial fibrosis is ventricular remodeling.

Therefore, LHF could reduce the expression of Col1al and Col3al in rat myocardium by inhibiting the expression of TGF-*β*1 and finally reduced the myocardial interstitial fibrosis.

VEGF is considered the most potent angiogenic factor. Some studies suggest that VEGF is beneficial to the heart by inhibiting fibrosis [26, 27], but mounting evidence indicates that overexpression of VEGF is harmful for the heart [28, 29]. VEGF is involved in cardiomyocyte hypertrophy and promotes ventricular remodeling [30]. In HF, the RASS system is activated and Ang II is synthesized and released in large quantities, which results in endothelial dysfunction and decreases the synthesis of NO. Endogenous NO exhibits an antimyocardial hypertrophy effect and prevents ventricular remodeling. A proper amount of VEGF increases the release of NO through the induction of eNOS. VEGF repairs damaged endothelial cells and inhibits the thickening of damaged endometrium, which improves ventricular remodeling. Hypertrophic cardiomyocytes in HF causes myocardial ischemia and hypoxia, which results in the high expression of VEGF [31]. The overexpression of VEGF disrupts the fat metabolism of the heart and causes cardiac hypertrophy, which forms of a vicious circle that eventually leads to HF [28]. VEGF receptor 2 (VEGFR2) is a member of the VEGFR superfamily and is expressed in the myocardial [32]. VEGF and its receptors combine to play a role in HF. Our study revealed decreased VEGF and VEGFR2 expression in the LHF groups (especially LHF-H group) and the perindopril group compared with the model group.

Therefore, LHF could enhance eNOS activity and decrease both VEGF and VEGFR2 expression. We hypothesized that LHF reversely inhibited VEGF/VEGFR2 signal pathway by stimulating eNOS expression.

In results, we found that each LHF group (especially LHF-H group) and the perindopril group showed a significantly reduced level of BNP in rats with HF. LHF-H group was significantly different in the other indicators compared with the model group. We concluded that the treatment effect of heart failure in the LHF-H group was better than that in the LHF-M and LHF-L groups.

## 5. Limitations

In this study, we found that LHF could enhance eNOS activity and decrease both VEGF and VEGFR2 expression. So we hypothesized that LHF reversely inhibited VEGF/VEGFR2 signal pathway by stimulating eNOS expression. However, we have not been able to study the pathway between eNOS and VEGF/VEGFR2 more deeply. In the future, we will clarify the cardioprotection of LHF more deeply.

## 6. Conclusions

In summary, the improvement of ventricular remodeling is an important part of delaying HF progression. Ventricular remodeling includes myocardial cell remodeling and myocardial interstitial remodeling. In the present study, we have demonstrated that LHF can prevent the progression of cardiac fibrosis and LV remodeling, which is possibly mediated by upregulating eNOS expression and downregulating Col1a1, Col3a1, TGF-*β*1, VEGF, and VEGFR2 expression. LHF could also attenuate cardiomyocyte apoptosis through downregulated caspase-3 expression to play a role in the cardioprotective effect. Although information on LHF is limited and further investigations are necessary to reveal the precise molecular mechanisms of action, our results provide a novel treatment option for HF.

## Figures and Tables

**Table 1:** Changes in body weight, heart weight, left ventricular weight, heart weight/body weight (HW/BW), and left ventricular weight/body weight (LVW/BW).

Group	*n*	Body weight (g)	Heart weight (mg)	Left ventricular weight (mg)	HW/BW (mg/g)	LVW/BW (mg/g)
Sham	8	520.13 ± 5.99	1251.19 ± 43.65	1047.59 ± 32.08	2.41 ± 0.20	2.01 ± 0.04
Model	7	493.00 ± 4.97	1489.22 ± 57.09	1212.19 ± 25.90	3.02 ± 0.09^Δ^	2.46 ± 0.03^Δ^
Perindopril	7	507.14 ± 3.85	1384.54 ± 21.26	1145.52 ± 24.61	2.73 ± 0.03^Δ*∗*^	2.26 ± 0.03^Δ*∗*^
LHF-H	8	503.25 ± 4.46	1399.20 ± 34.08	1161.63 ± 42.81	2.78 ± 0.04^Δ*∗*^^##^	2.31 ± 0.07^Δ*∗*^
LHF-M	7	504.43 ± 4.86	1433.47 ± 34.72	1200.07 ± 42.78	2.84 ± 0.04^Δ*∗*^^#★^	2.38 ± 0.06^Δ*∗*^^#^
LHF-L	6	501.67 ± 3.67	1407.26 ± 23.89	1174.83 ± 23.87	2.81 ± 0.03^Δ*∗*^^#▲^	2.34 ± 0.03^Δ*∗*^^#^

Notes: sham: control group; model: model group; perindopril: perindopril group; LHF-L: low-dose LHF group; LHF-M: medium-dose LHF group; LHF-H: high-dose LHF group. Data are expressed as mean ± standard deviation (SD). ^Δ^*P* < 0.01compared with sham; ^*∗*^*P* < 0.01 compared with model; ^#^*P* < 0.01, ^##^*P* < 0.05 compared with perindopril; ^*★*^*P* < 0.05 compared with LHF-H; ^*▲*^*P* < 0.05 compared with LHF-M. The analysis between groups of body weight and left ventricular weight accorded with normality and homogeneity of variance, so we used one-way ANOVA test. Body weight (*F*: 26.185, *P* < 0.01); left ventricular weight (*F*: 23.655, *P* < 0.01). The analysis between groups of heart weight, HW/BW, and LVW/BW did not conform to the normality and homogeneity of variance, so we used rank sum test of two independent samples and obtained the *Z* value. Heart weight (*P* < 0.01); HW/BW (*P* < 0.01); LVW/BW (*P* < 0.01).

**Table 2:** Changes in brain natriuretic peptide (BNP).

Group	*n*	BNP (ng/L)
Sham	8	393.95 ± 12.30
Model	7	785.83 ± 5.85^Δ^
Perindopril	7	511.81 ± 9.65^Δ*∗*^
LHF-H	8	539.40 ± 16.14^Δ*∗*^^#^
LHF-M	7	555.34 ± 12.40^Δ*∗*^^#★★^
LHF-L	6	567.33 ± 12.32^Δ*∗*^^#★^
*F*		828.289
*P*		<0.01

Notes: sham: control group; model: model group; perindopril: perindopril group; LHF-L: low-dose LHF group; LHF-M: medium-dose LHF group; LHF-H: high-dose LHF group. Data are expressed as mean ± standard deviation (SD). ^Δ^*P* < 0.01 compared with sham; ^*∗*^*P* < 0.01 compared with model; ^#^*P* < 0.01 compared with perindopril; ^★^*P* < 0.01, ^★★^*P* < 0.05 compared with LHF-H.

**Table 3:** Forward and reverse primer sequences used for real-time PCR.

Gene	Forward	Reverse
eNOS	5′CTTTCGGAAGGCGTTTGAC3′	5′AACTCTTGTGCTGCTCAGG3′
COL1A1	5′TCAAGATGTGCCACTCTG3′	5′ACCTTCGCTTCCATACTC3′
COL3A1	5′GTCCACAGCCTTCTACAC3′	5′TCCGACTCCAGACTTGAC3′
TGF-*β*1	5′AAGGACCTGGGTTGGAAGTG3′	5′TGGTTGTAGAGGGCAAGGAC3′
VEGF	5′GAGTCTGTGCTCTGGGATTTG3′	5′TCCTGCTACCTCTTTCCTCTG3′
VEGFR2	5′TTACTGTCCAGCCTGCTAC3′	5′CCAAAGAGCGTCCAAGTTC3′
GAPDH	5′GTCGGTGTGAACGGATTTG3′	5′TCCCATTCTCAGCCTTGAC3′

**Table 4:** eNOS, TGF-*β*1, VEGF, and VEGFR2 protein expression in myocardial tissue of different experimental groups.

Group	eNOS	TGF-*β*1	VEGF	VEGFR2
Sham	9561.96 ± 33.16	3133.25 ± 73.43	5203.72 ± 30.36	3537.75 ± 35.46
Model	1597.54 ± 17.20^Δ^	15123.09 ± 324.21^Δ^	12228.70 ± 265.10^Δ^	9925.97 ± 33.22^Δ^
Perindopril	5064.68 ± 53.09^Δ*∗*^	6719.59 ± 98.97^Δ*∗*^	9390.58 ± 194.44^Δ*∗*^	4661.49 ± 53.39^Δ*∗*^
LHF-L	1942.86 ± 28.25^Δ*∗*^^#★▲^	9283.95 ± 65.31^Δ*∗*^^#★▲^	12163.34 ± 319.95^Δ#★▲^	8712.10 ± 31.46^Δ*∗*^^#★▲^
LHF-M	5920.88 ± 33.99^Δ*∗*^^#★^	4994.44 ± 94.47^Δ*∗*^^#★^	8713.85 ± 176.83^Δ*∗*^^#★^	5092.42 ± 63.87^Δ*∗*^^#★^
LHF-H	7623.87 ± 37.84^Δ*∗*^^#^	3805.52 ± 53.03^ΔΔ*∗*^^#^	7762.68 ± 130.11^Δ*∗*^^#^	3571.35 ± 108.94^*∗*^^#^
*F*	23205.479	2848.484	502.776	6063.123
*P*	<0.01	<0.01	<0.01	<0.01

Notes: sham: control group; model: model group; perindopril: perindopril group; LHF-L: low-dose LHF group; LHF-M: medium-dose LHF group; LHF-H: high-dose LHF group. Data are expressed as mean ± standard deviation (SD). ^Δ^*P* < 0.01, ^ΔΔ^*P* < 0.05 compared with sham; ^*∗*^*P* < 0.01 compared with model; ^#^*P* < 0.01 compared with perindopril; ^★^*P* < 0.01 compared with LHF-H; ^▲^*P* < 0.01 compared with LHF-M.

**Table 5:** Caspase-3 protein expression in myocardial tissue of different experimental groups.

Group	Caspase-3
Sham	7970.00 ± 768.09
Model	43367.67 ± 2851.72^Δ^
Perindopril	37305.67 ± 1761.21^Δ*∗∗*^
LHF-H	17494.33 ± 5575.60^Δ*∗*^^#^
LHF-M	38680.00 ± 1566.17^Δ★^
LHF-L	45671.67 ± 1120.39^Δ#★▲▲^
*F*	90.841
*P*	<0.01

Notes: sham: control group; model: model group; perindopril: perindopril group; LHF-L: low-dose LHF group; LHF-M: medium-dose LHF group; LHF-H: high-dose LHF group. Data are expressed as mean ± standard deviation (SD). ^Δ^*P* < 0.01 compared with sham; ^*∗*^*P* < 0.01, ^*∗∗*^*P* < 0.05 compared with model; ^#^*P* < 0.01 compared with perindopril; ^*★*^*P* < 0.01 compared with LHF-H; ^▲▲^*P* < 0.05 compared with LHF-M.

**Table 6:** Col1a1, Col3a1, eNOS, TGF-*β*1, VEGF, and VEGFR2 relative expression in myocardial tissue of experimental different groups.

Group	Col1a1	Col3a1	eNOS	TGF-*β*1	VEGF	VEGFR2
Sham	0.015 ± 0.002	0.010 ± 0.001	0.055 ± 0.003	0.020 ± 0.002	0.014 ± 0.001	0.004 ± 0.000
Model	0.080 ± 0.011^Δ^	0.037 ± 0.002^Δ^	0.019 ± 0.001^Δ^	0.124 ± 0.017^Δ^	0.063 ± 0.008^Δ^	0.015 ± 0.001^Δ^
Perindopril	0.029 ± 0.003^ΔΔ*∗*^	0.016 ± 0.001^Δ*∗*^	0.039 ± 0.003^Δ*∗*^	0.047 ± 0.009^Δ*∗*^	0.040 ± 0.004^Δ*∗*^	0.007 ± 0.001^Δ*∗*^
LHF-L	0.063 ± 0.006^Δ*∗*^^#★▲^	0.031 ± 0.001^Δ*∗*^^#★▲^	0.026 ± 0.002^Δ*∗*^^#★▲▲^	0.092 ± 0.007^Δ*∗*^^#★▲^	0.051 ± 0.003^Δ*∗∗*^^##★▲▲^	0.012 ± 0.001^Δ*∗*^^#★▲^
LHF-M	0.045 ± 0.007^Δ*∗*^^#★^	0.023 ± 0.001^Δ*∗*^^#★^	0.030 ± 0.002^Δ*∗*^^#★^	0.067 ± 0.005^Δ★^^∗##^	0.042 ± 0.005^Δ*∗*★★^	0.009 ± 0.001^Δ*∗*^^##★★^
LHF-H	0.022 ± 0.001^*∗*^	0.014 ± 0.001^Δ*∗*^	0.044 ± 0.003^Δ*∗*^^##^	0.037 ± 0.005^ΔΔ*∗*^	0.031 ± 0.006^Δ*∗*^	0.006 ± 0.000^Δ*∗*^
*F*	51.979	191.059	83.422	56.008	34.994	81.270
*P*	<0.01	<0.01	<0.01	<0.01	<0.01	<0.01

Notes: sham: control group; model: model group; perindopril: perindopril group; LHF-L: low-dose LHF group; LHF-M: medium-dose LHF group; LHF-H: high-dose LHF group. Data are expressed as mean ± standard deviation (SD). ^Δ^*P* < 0.01, ^ΔΔ^*P* < 0.05 compared with Sham; ^*∗*^*P* < 0.01, ^*∗∗*^*P* < 0.05 compared with model; ^#^*P* < 0.01, ^##^*P* < 0.05 compared with perindopril; ^★^*P* < 0.01, ^★★^*P* < 0.05 compared with LHF-H; ^▲^*P* < 0.01, ^▲▲^*P* < 0.05 compared with LHF-M.

## Data Availability

The data used to support the findings of this study are available from the corresponding author upon request.

## Supplementary Materials

Supplementary Materials Graphical abstract.
