# Supplementary figures and images for: Luhong Formula Has a Cardioprotective Effect on Left Ventricular Remodeling in Pressure-Overloaded Rats
Source: Evid Based Complement Alternat Med. 2020 May 30;2020:4095967. doi: 10.1155/2020/4095967 (PMC7277070; doi:10.1155/2020/4095967)

Graphical Abstract

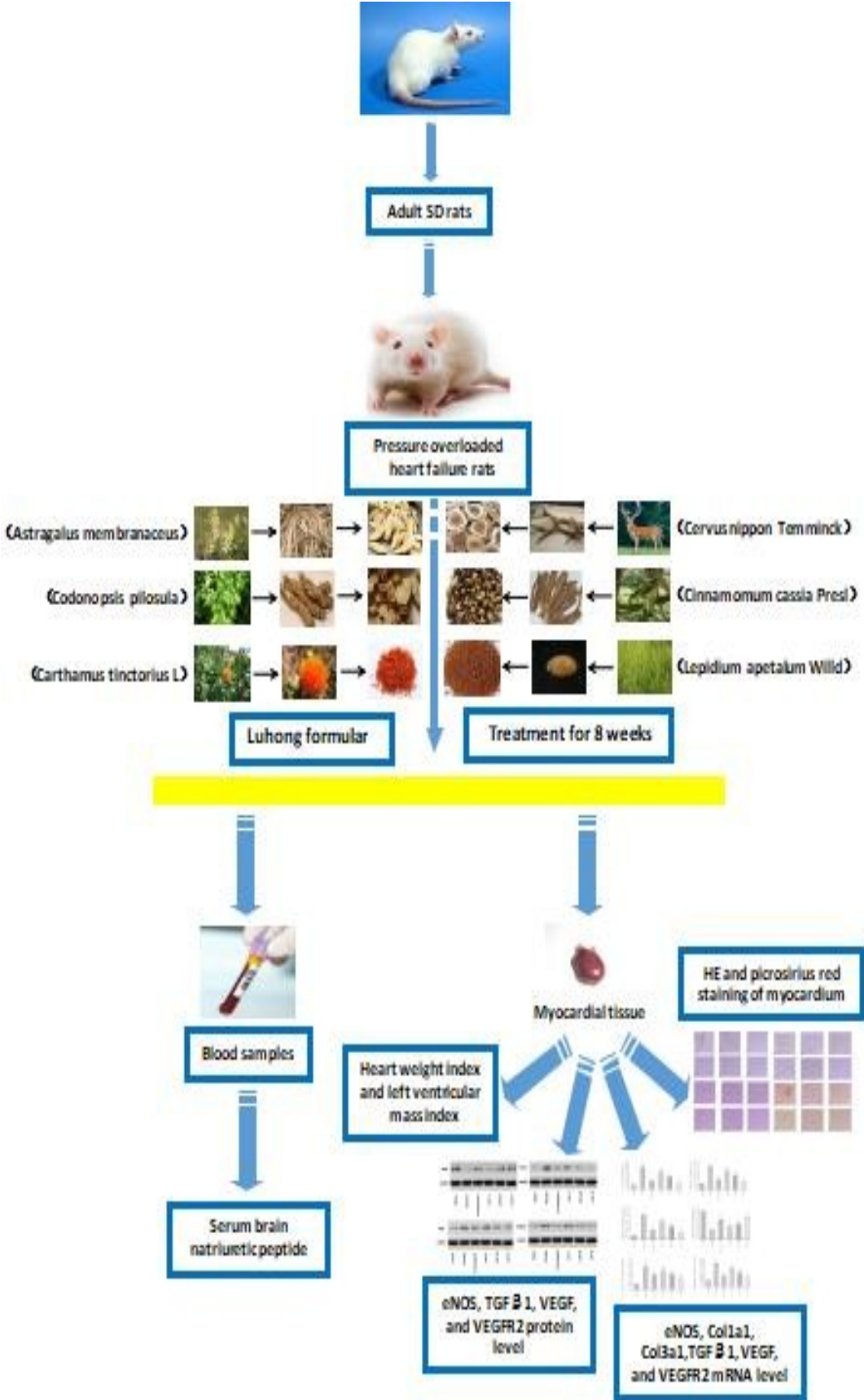

Supplement: Supplementary Materials — Graphical abstract. [file 4095967.f1.pdf]
